# The value of miR-510 in the prognosis and development of colon cancer

**DOI:** 10.1515/med-2021-0251

**Published:** 2021-05-15

**Authors:** Junjie Hang, Feifei Wei, Zhiying Yan, Xianming Zhang, Kequn Xu, Yingwei Zhu

**Affiliations:** Department of Oncology, The Affiliated Changzhou No. 2 People’s Hospital of Nanjing Medical University, Changzhou, Jiangsu, 213003, People’s Republic of China

**Keywords:** colon cancer, miR-510, prognosis, cell proliferation, migration

## Abstract

**Purpose:**

Colon cancer is one of the malignant tumors that threatens human health. miR-510 was demonstrated to play roles in the progression of various cancers; its dysregulation was speculated to be associated with the development of colon cancer.

**Methods:**

One hundred and thirteen colon cancer patients participated in this research. With the help of RT-qPCR, the expression of miR-510 in collected tissues and cultured cells was analyzed. The association between miR-510 expression level and clinical features and prognosis of patients was evaluated. Moreover, the effects of miR-510 on cell proliferation, migration, and invasion of colon cancer were assessed by CCK8 and Transwell assay.

**Results:**

miR-510 significantly upregulated in colon cancer tissues and cell lines relative to the adjacent normal tissues and colonic cells. The expression of miR-510 was significantly associated with the TNM stage and poor prognosis of patients, indicating miR-510 was involved in the disease progression and clinical prognosis of colon cancer. Additionally, the upregulation of miR-510 significantly promoted cell proliferation, migration, and invasion of colon cancer, while its knockdown significantly inhibited these cellular processes. SRCIN 1 was the direct target of miR-510 during its promoted effect on the development of colon cancer.

**Conclusion:**

The upregulation of miR-510 acts as an independent prognostic indicator and a tumor promoter by targeting SRCIN 1 in colon cancer, which provides novel therapeutic strategies for colon cancer.

## 1 Introduction

Colon cancer ranks at a top place in the worldwide rankings of cancer incidence and mortality with the increasing number of new cases [1,2]. In China, the incidence of CRC was 33.1 million and the mortality was 15.9 million, according to the cancer statistical data in 2016 [3]. The rapid tumor progression and high rate of recurrence result in a poor prognosis and high mortality rate of colon cancer [4,5]. Oncogenes and tumor suppressors play vital roles in the occurrence and development of colon cancer [6,7,8]. With the increasing availability of therapeutic options for colon cancer, efficient biomarkers have become clinically relevant to predicate the prognosis of patients and the tumor development of colon cancer [9]. Although a variety of tumor regulators of various cancers have been identified, there is still an urgent need to develop optimal markers for the prognosis and progression of colon cancer.

MicroRNAs are endogenous noncoding RNAs with a length of about 18–25 nucleotides, which are involved in the regulation of mRNA translation and degradation by binding the 3′ untranslated region of the target gene mRNA [10,11,12]. Accumulating studies demonstrated the biological mechanism of colon cancer, identifying a number of miRNAs correlated with the occurrence, development, migration, and recurrence of various cancers including colon cancer [13,14]. Numerous differently expressed miRNAs in colon cancer have been reported to be closely related to the biological and clinical characteristics of colon cancer and play roles in its prognosis and progression. For example, downregulated miR-378 inhibits the proliferation, migration, and invasion of colon cancer and serves as a potential target for the treatment of colon cancer [7]. It is necessary to explore novel miRNAs in colon cancer, which can provide more therapeutic strategies for colon cancer.

miR-510 is a dysregulated miRNA in colon cancer and it also has been reported to be differentially expressed and play roles in the tumor progression of many other cancers, such as non-small cell lung cancer (NSCLC), thyroid cancer, breast cancer, and renal cell carcinoma [15,16,17,18,19]. Therefore, it is speculated that the dysregulation of miR-510 in colon cancer might indicate the potential functional role in the development and progression of colon cancer. Here, the expression of miR-510 was investigated in colon cancer tissues and cells. The association of miR-510 expression with the clinical prognosis and features of patients was also evaluated. Besides, the role of miR-510 in the proliferation, migration, and invasion of colon cancer cells was also assessed.

## 2 Materials and methods

### 2.1 Colon cancer patients and tissue samples

One hundred and thirteen paired colon cancer tissues and adjacent normal tissues were collected from patients with colon cancer who underwent surgery in The Affiliated Changzhou No.2 People’s Hospital of Nanjing Medical University from 2012 to 2014. The isolated samples were confirmed with pathology diagnosis following the International Union against Cancer (UICC). Informed consent was obtained from each patient and this study obtained ethical approval from the ethics committee of The Affiliated Changzhou No. 2 People’s Hospital of Nanjing Medical University. All samples were maintained in liquid nitrogen and stored at −80°C until use. A 5-year follow-up survey was conducted to obtain the survival information of patients. The clinical features of the patients are summarized in Table 1.

**Table 1:** Association between miR-510 expression level and the clinical features of colon patients

	Total patients (*n* = 113)	miR-510 expression level		*P* value
		Low (*n* = 48)	High (*n* = 65)	
**Age**				0.535
≤60	58	30	28	
>60	55	18	37	
**Gender**				0.593
Male	63	32	31	
Female	50	16	34	
**Tumor size (cm)**				0.143
≤5	60	22	38	
>5	53	26	27	
**TNM stage**				0.031
I–II	75	39	36	
III–IV	38	9	29	
**Differentiation**				0.164
Well and moderate	72	38	34	
Poor	41	10	31	
**Lymph node metastasis**				0.036
Negative	80	38	42	
Positive	33	10	23	

### 2.2 Cell culture and cell transfection

The human colon cancer cells, LoVo, CaCo-2, SW480, and HCT-116, and normal colonic mucosa cell NCM460 were obtained from Shanghai Cell Bank and cultured in DMEM medium (Thermo Fisher Scientific) with 10% fetal bovine serum (FBS) at 37°C in a 5% CO_2_ humidified incubator.

miR-510 mimic, miR-510 inhibitor, and corresponding negative controls (mimic NC and inhibitor NC) were purchased from RiboBio (Guangzhou, China) for the overexpression or knockdown of miR-510. The sequences were: 5′-UACUCAGGAGAGUGGCAAUCAC-3′ for miR-510 mimic, 5′-GUGAUUGCCACUCUCCUGAGUA-3′ for the miR-510 inhibitor, 5′- GGACCAAATCTCGAGATTTGG-3′ for mimic NC, 5′-UUCUCCGAACGUGUCACGUU-3′ for inhibitor NC. Cell transfection was performed with the Lipofectamine 2000 Reagent (Invitrogen, USA) following the manufacturers’ protocol.

### 2.3 Quantitative real-time PCR (RT-qPCR)

Total RNA was extracted from collected tissues and cultured cells with the TRIzol reagent (Invitrogen, Carlsbad, CA, USA). Extracted RNA was reverse-transcribed to cDNA with the Revert Aid First Strand cDNA Synthesis Kit (Thermo Scientific). To quantify miR-510, RT-qPCR was performed with the SYBR Green I Master Mix kit (Invitrogen) and the 7300 Real-Time PCR System (Applied Biosystems, USA) in triplicate. U6 was used as an internal control and the 2^−ΔΔCt^ method was used to calculate the relative expression of miR-510.

### 2.4 CCK8 proliferation assay

Cells were seeded into 96-well plates at 5 × 10^3^ cells per well. Cell counting kit-8 (CCK-8) reagent (Dojindo, Kumamoto, Japan) was diluted as protocol and added to each well at 0, 24, 48, and 72 h and continue incubating for 4 h at 37°C with 5% CO_2_. Absorbance at 450 nm was measured with a microplate reader (Thermo Fisher Scientific). Experiments were conducted in triplicate and obtained the mean values.

### 2.5 Transwell migration and invasion assay

The 24-well transwell chambers with a pore size of 8 µm polycarbonate membrane (Multiskan MK3, Thermo, Waltham, MA, USA) were used to analyze the migration and invasion capacities of colon cells. Cells were seeded into the upper chamber with a serum-free medium without Matrigel coating at a density of 1 × 10^5^ cells per well for the migration assay. For the invasion assay, the upper chamber was coated with Matrigel (BD Biosciences, Franklin Lakes, NJ, USA). Culture medium with 10% FBS was placed in the lower chamber as a chemoattractant. The chambers were incubated at 37°C for 48 h, then the migrated and invasive cells were stained with 0.05% crystal violet for 30 min and counted by the microscope.

### 2.6 Dual-luciferase reporter assay

The potential targets were first predicted online (http://www.targetscan.org) and the binding sites were also obtained. The 3′UTR of SRCIN 1 containing the putative binding sites was cloned into the dual-luciferase reporter vector pmirGLO (SRCIN 1 WT, Promega Corporation, USA), while the mutant vectors were also constructed with mutations in the binding sites (SRCIN 1 MT).

LoVo cells were seeded 24-well plates and transfected with pmirGLO-SCRIN1 WT or pmirGLO-SRCIN 1 MT together with miR-510 mimics or inhibitor or negative controls. Then, the relative luciferase activity was measured by the Dual-Luciferase Reporter Assay Kit (Promega Corporation, USA) according to the manufacturer’s instructions.

### 2.7 Statistical analysis

Data are expressed as mean value ± standard deviation (SD) and analyzed by the SPSS 20.0 software (SPSS, Inc., Chicago, IL, USA) and GraphPad Prism 5.0 software (GraphPad Software, Inc., Chicago, USA). One-way ANOVA and paired student’s *t*-test were employed for the comparison between groups. Kaplan–Meier analysis and Cox regression analysis were used to determine the prognostic value of miR-510 in colon cancer. *P* < 0.05 was considered to be statistically significant.

## 3 Results

### 3.1 miR-510 is expressed at a high level in colon cancer tissues and cell lines

**Figure 1:**
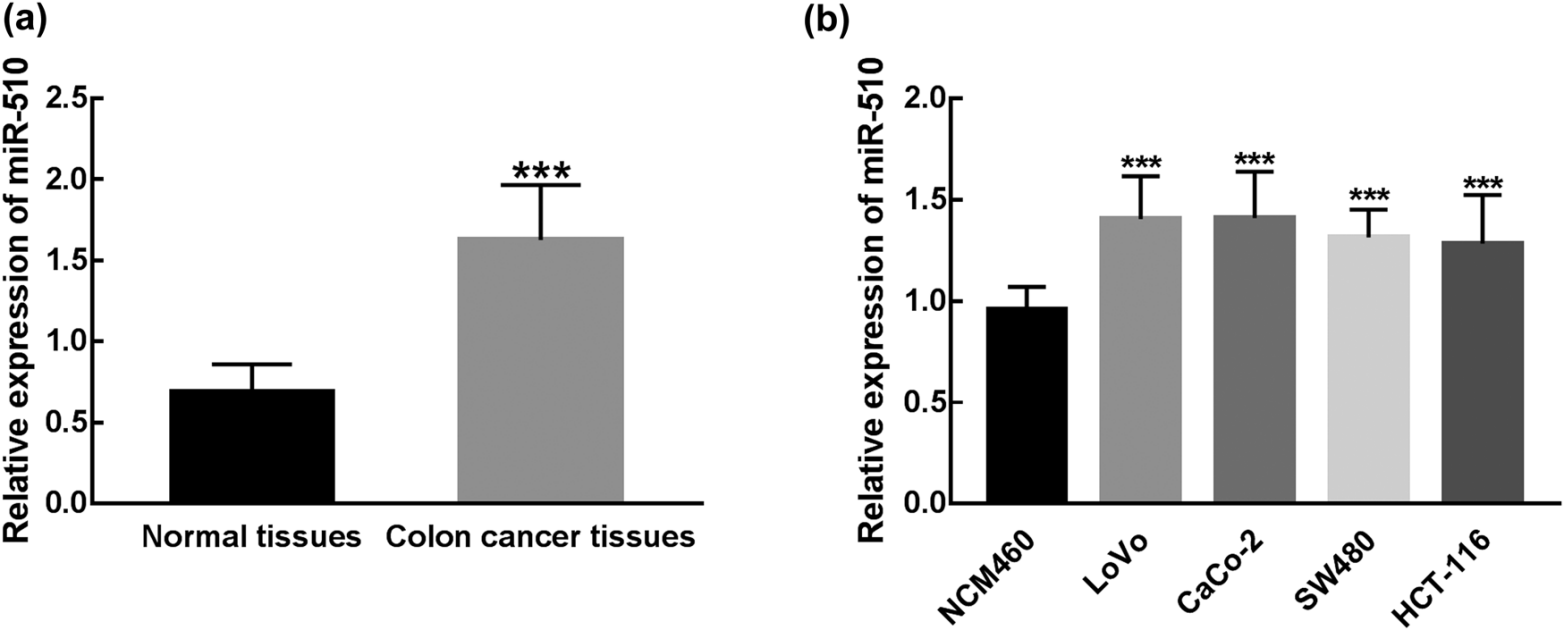
The expression of miR-510 in collected tissues and cultured cell lines. (a) miR-510 was significantly upregulated in colon cancer tissues compared with that in adjacent normal tissues (***P < 0.001). (b) miR-510 was significantly upregulated in colon cancer cell lines (LoVo, CaCo-2, SW480, and HCT-116) compared with normal colonic mucosa cells (NCM460) (***P < 0.001).

The expression of miR-510 in collected tissues and cultured cells was detected by RT-qPCR. A significant increase in the expression of miR-510 was observed in colon tissues, compared with apparently adjacent normal tissues (*P* < 0.001, Figure 1a). Similarly, in colon cells (LoVo, CaCo-2, SW480, and HCT-116), miR-510 was significantly upregulated relative to the expression in NCM460, a normal colonic mucosa cell (*P* < 0.001, Figure 1b).

### 3.2 miR-510 is associated with the TNM stage of colon cancer patients

With the mean value of the miR-510 expression in colon cancer tissues as the cutoff, 113 patients were divided into a miR-510 low expression group and a miR-510 high expression group. The association between miR-510 expression and patients’ clinical features was evaluated. A significant association between miR-510 expression and the TNM stage (*P* = 0.031) and lymph node metastasis (*P* = 0.036) of colon cancer patients was observed, while age, gender, tumor size, and differentiation of patients showed no significant relationship with the miR-510 expression, indicating the involvement of miR-510 in the disease progression of colon cancer (*P* > 0.05, Table 1).

### 3.3 miR-510 serves as an independent indicator for the prognosis of colon cancer patients

**Figure 2:**
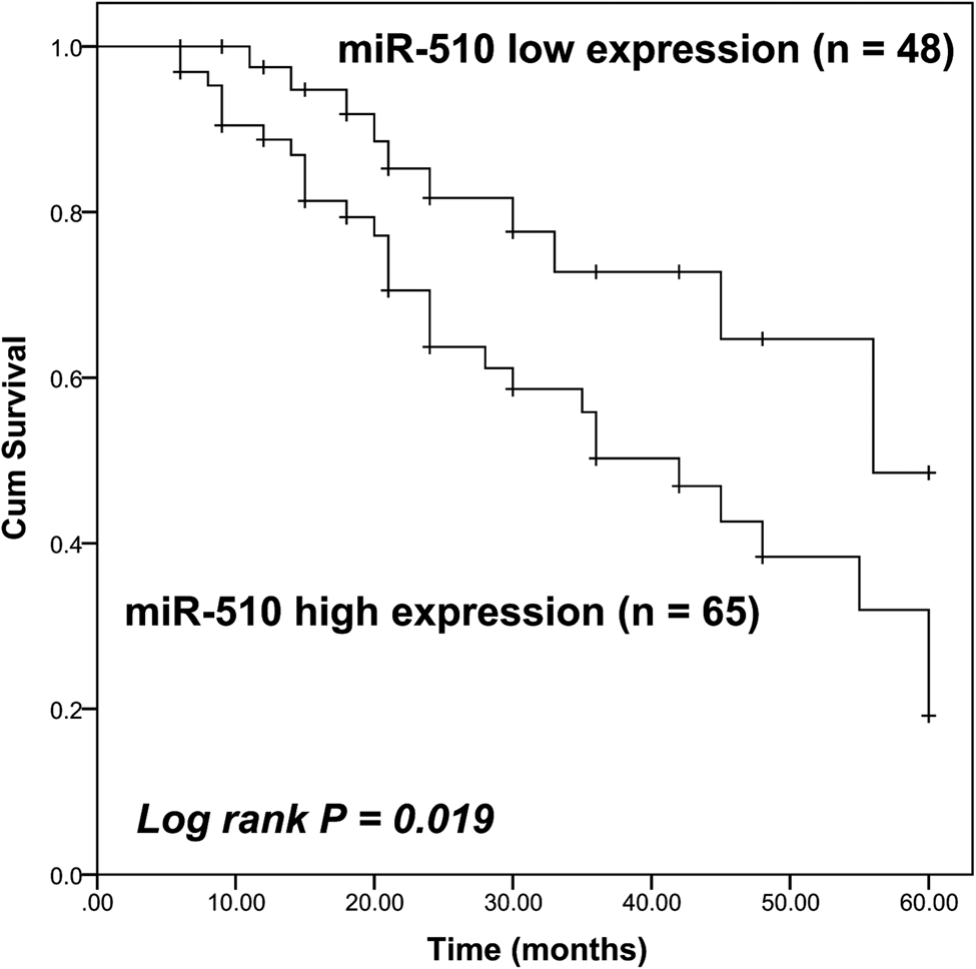
Kaplan–Meier curve of colon cancer patients with different miR-510 expression levels. Colon patients were divided into the high miR-510 expression group and the low miR-510 expression group according to the mean value of the miR-510 expression in colon cancer tissues. Patients with high miR-510 expression levels had a poorer prognosis than that of patients with low miR-510 expression levels (Log-rank *P* = 0.019).

The Kaplan–Meier curve showed that the survival rate of colon cancer patients with high miR-510 expression was poorer than that of patients with low miR-510 expression (Log-rank *P* = 0.019, Figure 2). The prognostic value of miR-510 was further assessed by the Cox regression analysis shown in Table 2. It was found that the miR-510 expression level (95% CI = 1.476–7.204, *P* = 0.003) and the TNM stage of patients (95% CI = 1.034–5.267, *P* = 0.041) were considered as two independent indicators for the prognosis of colon patients with the HR value of 3.261 and 2.333, respectively.

**Table 2:** Cox regression analysis on the association between clinical features and survival rate of patients

	HR factor	95% CI	*P* value
miR-510	3.261	1.476–7.204	0.003
Age	1.261	0.637–2.495	0.506
Gender	1.184	0.576–2.433	0.646
Tumor size	1.450	0.686–3.063	0.330
TNM stage	2.333	1.034–5.267	0.041
Differentiation	1.439	0.720–2.876	0.302
Lymph node metastasis	2.002	0.745–5.382	0.169

### 3.4 miR-510 acts as a tumor promoter of colon cancer

**Figure 3:**
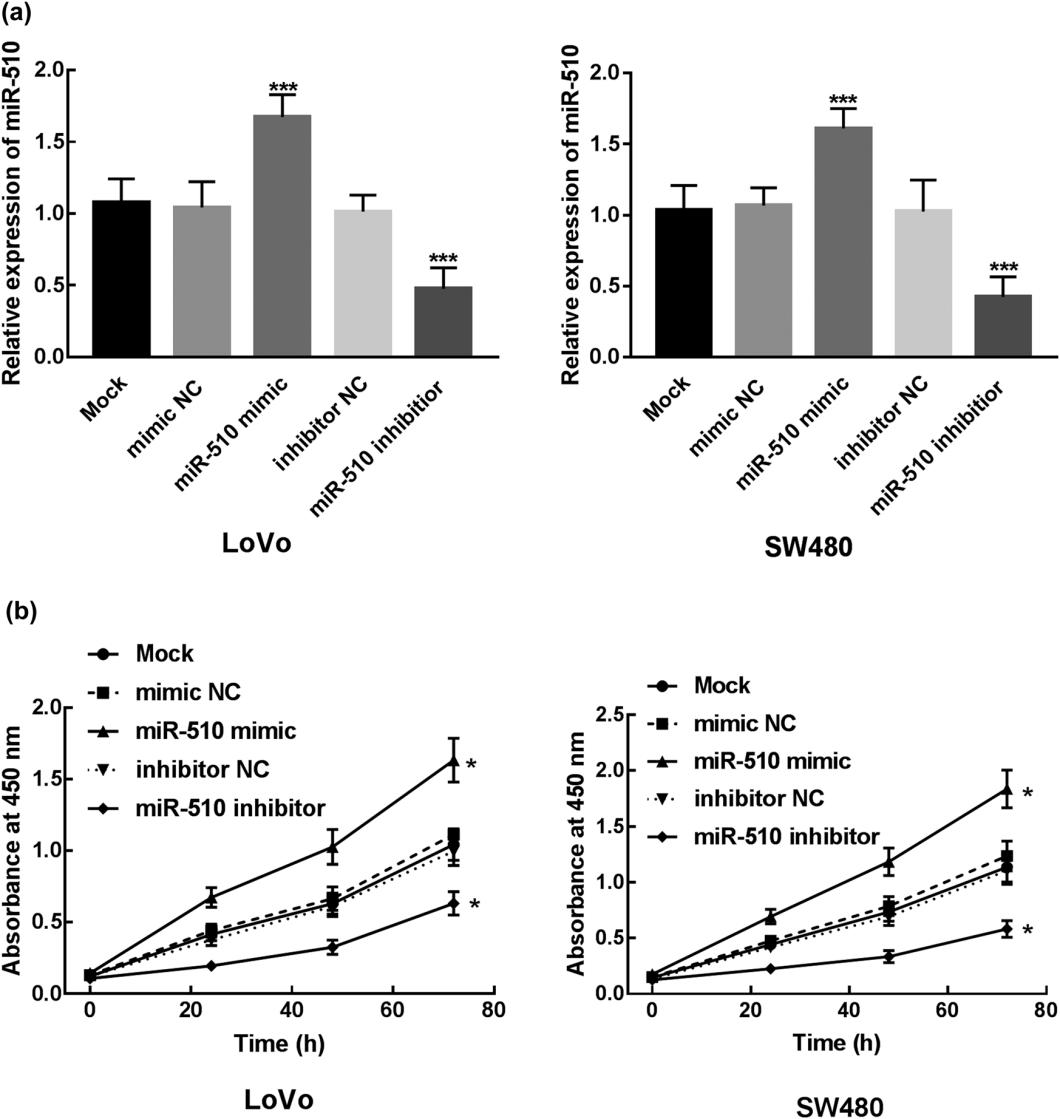
Cell transfection and its effect on the proliferation of colon cancer cells. (a) Transfection of miR-510 mimics significantly increased the expression of miR-510 in LoVo and SW480 cells, while the transfection of miR-510 inhibitor significantly decreased the expression of miR-510 in LoVo and SW480 cells, indicating the transfection was successful (***P < 0.001). (b) Overexpression of miR-510 significantly enhanced the proliferation of LoVo and SW480 cells, while the knockdown of miR-510 significantly inhibited the proliferation of LoVo and SW480 cells (*P < 0.05).

miR-510 mimic, miR-510 inhibitor, mimic NC, and inhibitor NC were transfected into LoVo and SW480 to overexpress or knockdown the expression of miR-510 in colon cancer cells. The expression of miR-510 was significantly decreased after the transfection of miR-510 inhibitor and increased after the transfection of miR-510 mimic (*P* < 0.001, Figure 3a).

**Figure 4:**
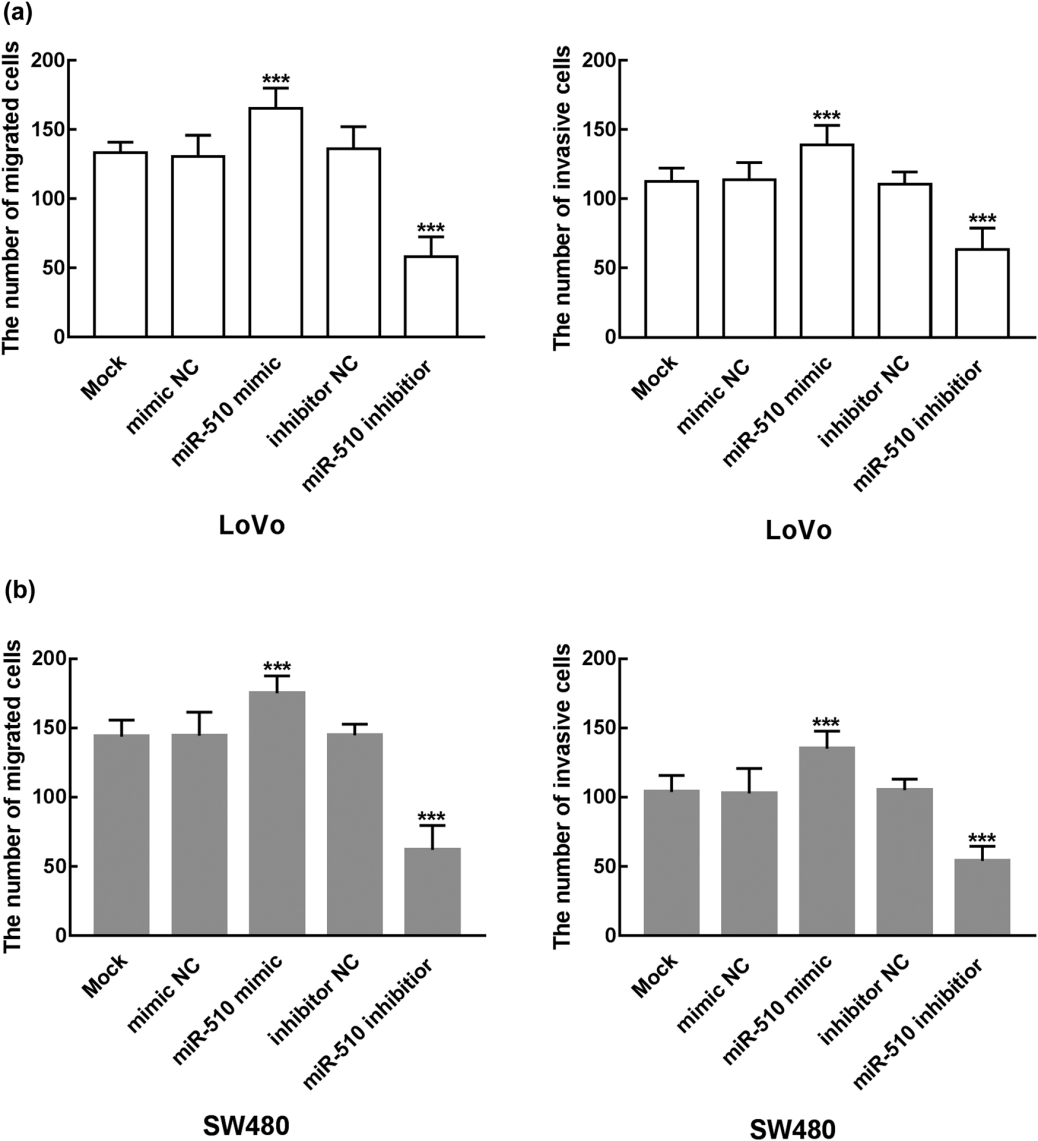
Effect of miR-510 expression level on cell migration and invasion of LoVo and SW480 cells. (a) The overexpression of miR-510 significantly enhanced cell migration and invasion of LoVo cells, while the knockdown of miR-510 significantly inhibited cell migration and invasion of LoVo cells (***P < 0.001). (b) The overexpression of miR-510 significantly enhanced cell migration and invasion of SW480 cells, while the knockdown of miR-510 significantly inhibited cell migration and invasion of SW480 cells (***P < 0.001).

The proliferation of transfected cells was analyzed by CCK8 assay. As shown in Figure 3b, the proliferation ability of LoVo and SW480 was promoted by the transfection of miR-510 mimic and inhibited by the transfection of miR-510 inhibitor, which indicated the enhanced effect of miR-510 on the proliferation of colon cancer cells (*P* < 0.05). For the migration and invasion of colon cancer cells, the Transwell assay was employed. The migration and invasion of LoVo were significantly increased after the transfection of miR-510 mimic, while the transfection of miR-510 inhibitor significantly inhibited cell migration and invasion of LoVo (*P* < 0.001, Figure 4a). Likewise, the number of migrated and invasive cells of SW480 was enhanced by the overexpression of miR-510 and suppressed by the knockdown of miR-510 (*P* < 0.001, Figure 4b).

### 3.5 SRCIN 1 is the direct target of miR-510

**Figure 5:**
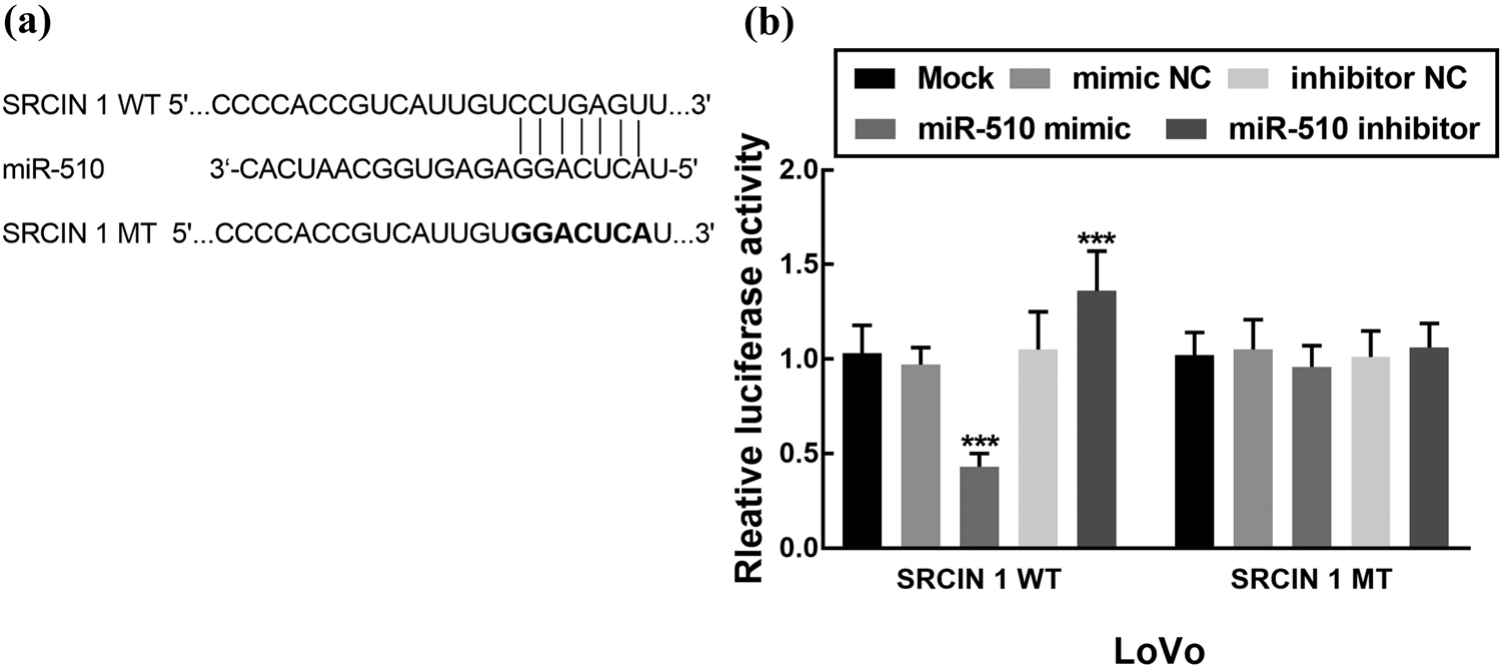
SRCIN 1 is the direct target of miR-510. (a) The relative luciferase activity of SRCIN 1 WT was significantly inhibited by the overexpression of miR-510 and enhanced by the silencing of miR-510 (***P < 0.001).

SRCIN 1 was predicted as the potential target of miR-510 by online biology software. The binding sites between SRCIN 1 WT and miR-510 were shown in Figure 5a. Further, the interaction between miR-510 and SRCIN 1 was evaluated by the dual-luciferase reporter assay. It was found that the relative luciferase activity of SRCIN 1 WT was inhibited by the overexpression of miR-510 significantly and enhanced by the knockdown of miR-510 (*P* < 0.001, Figure 5b). However, the dysregulation of miR-510 did not suppress or promote the relative luciferase activity of SRCIN 1 MT (*P* > 0.05, Figure 5b).

## 4 Discussion

Human colon cancer is one of the leading causes of cancer-related death worldwide [20]. Identification of cancer-specific biomarkers can help understand the tumor progression and development and provide more novel therapeutic strategies [21]. With the development of molecular biology, miRNAs have been demonstrated to be involved in cell proliferation, differentiation, and apoptosis of various diseases including cancers, which are closely related to the tumor occurrence, development, and prognosis [22,23,24]. For example, the downregulation of miR-210 in pancreatic cancer could promote cell proliferation and tumor progression by regulating the expression of E2F3 [25].

Previously, numerous aberrantly expressed miRNA in colon cancer have been identified and reported to be closely correlated with the prognosis and clinical features of colon cancer patients. For instance, miR-4709 was highly expressed in colon cancer and closely associated with the death and overall survival of patients indicating the significant prognostic role of miR-4709 in colon cancer [26]. The downregulation of miR-21 and the upregulation of miR-138 in colon cancer were found to be closely correlated with differentiation, lymph node metastasis, TNM stage, and distant metastasis of patients [27].

By comparing the normal tissues and colon cancer tissues, miR-510 was found to be significantly upregulated in colon cancer and the similar upregulation of miR-510 was also observed in colon cancer cells compared with normal colonic mucosa cells. Consistently, miR-510 was identified to be upregulated in both microsatellite stable (MSS) and microsatellite instable (MSI) cancers of colon cancer [19]. Therefore, this kind of dysregulation of miR-510 makes it possible to act as a biomarker in the progression and prognosis of colon cancer. Additionally, the miR-510 expression level was significantly associated with the TNM stage of patients and the high miR-510 expression level predicated a poor prognosis of colon cancer patients, which suggested the involvement of miR-510 in the disease progression of colon cancer and the prognostic biomarker role of miR-510 in colon cancer. Other clinical features of patients, such as tumor size, differentiation, and lymph node metastasis status, were also vital factors that correlated with the prognosis of patients [28,29,30]. However, there was no significant association found between these clinical features and the survival rate of patients. This may result from the small sample size of this study, which is a limitation of this study. Therefore, a larger sample size is needed in further studies.

Previous studies also reported the biological functions of differently expressed miRNAs in various cancers including colon cancer. miR-137 acts as a tumor suppressor, which inhibits the proliferation, migration, and invasion of colon cancer cells by targeting TCF4 [31]. miR-510 was also revealed to participate in the progression of many other cancers. In thyroid cancer, the overexpression of miR-510 was found to promote cell proliferation, migration, and invasion of thyroid cancer cells by suppressing SNHG15 [16]. Similarly, miR-510 was upregulated in breast cancer that increases cell growth, migration, invasion, and colony formation of breast cancer [17]. Conversely, miR-510 acts as a tumor suppressor in renal cell carcinoma as its inhibitory effect on cell proliferation and migration and enhanced effect on cell apoptosis [18]. In colon cancer investigated in this research, overexpression of miR-510 promoted cell proliferation, migration, and invasion of colon cancer, while the knockdown of miR-510 inhibited those cellular processes indicating that miR-510 acts as a tumor promoter miRNA in the progression of colon cancer. Moreover, SRCIN 1 was identified as the direct target of miR-510, as the overexpression of miR-510 dramatically suppressed the activity of SRCIN 1. Previously, SRCIN 1 was revealed to serve as the direct target of miR-510 in NSCLC, by regulating which miR-510 promoted the development of NSCLC [15]. Therefore, it was speculated that miR-510 promoted the proliferation, migration, and invasion of colon cancer cells by targeting SRCIN 1.

To conclude, the upregulation of miR-510 in colon cancer was significantly associated with the TNM stage and poor prognosis of patients. In colon cancer cells, the miR-510 upregulation significantly promoted cell proliferation, migration, and invasion of colon cancer by targeting SRCIN 1. These results indicated the involvement of miR-510 in the progression of colon cancer and it can also serve as a prognostic biomarker for colon cancer, which provides a novel potential therapeutic target for colon cancer.

## Figures and Tables

**Figure A1:**
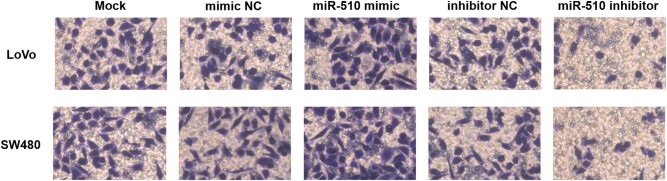
Representative images of the migration of LoVo and SW480 cells by the Transwell assay.

**Figure A2:**
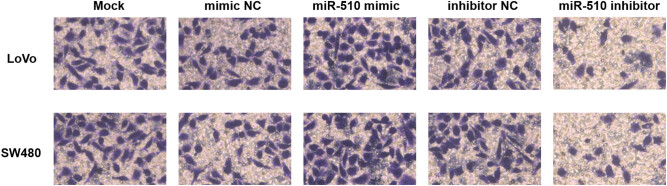
Representative images of the invasion of LoVo and SW480 cells by the Transwell assay.
